# Determination and Profiling of Human Skin Odors Using Hair Samples

**DOI:** 10.3390/molecules24162964

**Published:** 2019-08-15

**Authors:** Diva S. Tavares, Paulo R. R. Mesquita, Vanessa R. Salgado, Frederico de Medeiros Rodrigues, José Carlos Miranda, Manoel Barral-Netto, Jailson B. de Andrade, Aldina Barral

**Affiliations:** 1 Faculdade de Medicina do Centro Universitário Christus (UNICHRISTUS), Fortaleza, CE 60190-060, Brazil; 2 Instituto Gonçalo Moniz (IGM)–Fundação Oswaldo Cruz (FIOCRUZ), Salvador, BA 40296-710, Brazil; 3 Instituto de Química da Universidade Federal da Bahia (UFBA), Salvador, BA 40170-115, Brazil; 4 Programa de Pós-graduação em Desenvolvimento Regional e Meio Ambiente, Faculdade Maria Milza (FAMAM), Governador Mangabeira, BA 44350-000, Brazil; 5 Faculdade de Medicina Veterinária da União Metropolitana de Educação e Cultura (UNIME), Lauro de Freitas, BA 42700-000, Brazil; 6 Faculdade de Medicina da Universidade Federal da Bahia (UFBA), Salvador, BA 40026-010, Brazil; 7 Instituto Nacional de Ciência e Tecnologia de Investigação em Imunologia (iii-INCT), São Paulo, SP 05403-900, Brazil; 8 Instituto Nacional de Ciência e Tecnologia em Energia e Ambiente (INCT-EA), Salvador, BA 40170-115, Brazil

**Keywords:** HS-SPME/GC-MS, human skin odors, volatile organic compounds

## Abstract

**Background.** There is no gold standard method for human skin odor determination; several techniques can be applied to collect, extract, transfer, and detect human skin odors. However, none of these methods are suitable for field sampling of a large number of individuals. **Objective**. The present study aimed to develop a simple, fast, non-invasive, and low-cost method for such a purpose. **Methods.** Considering that hair from legs can act as a retention mesh of volatile organic compounds (VOCs), samples of leg hairs provided by healthy adult males were collected and solid-phase microextraction (SPME), in headspace (HS) mode, coupled to gas chromatography (GC) and mass spectrometry (MS) analysis of the samples was carried out. A pilot test was applied to detect five quality markers that are frequently reported in human skin odors. Then, several steps were performed for method standardization. The method was applied to 36 different individuals (3 sampled under laboratory conditions and 33 under field conditions), aiming to evaluate its applicability in both environments. **Findings.** A total of 49 VOCs were identified, and 73.5% of these have been reported in previous studies. **Main Conclusions.** Hair from legs can be considered an efficient tool for human skin odor sampling and a suitable and practical matrix for human skin odor profile determination by using HS-SPME/GC-MS.

## 1. Introduction

Human odors have been investigated for a range of purposes, such as for human pheromone identification or the investigation of volatile biomarkers in breath, which can be used as a non-invasive and low-cost diagnostic tool for many diseases [1,2]. Many studies have focused on human skin odors and their relation to the attraction of insects harboring human pathogens, aiming to understand the relationship between vectors and hosts and aid in the development of alternative methods for vector-borne disease control [1,3,4,5].

There is no gold standard method for human skin odor determination; many techniques can be applied to collect, extract, transfer, and detect human skin odors [6]. However, solid-phase microextraction (SPME) in headspace (HS) mode coupled to gas chromatography (GC) and mass spectrometry (MS), hereafter referred to as HS-SPME/GC-MS, has been successfully used for the confinement, extraction, separation, and detection of volatile organic compounds (VOCs) that constitute such odors [7,8,9]. 

The first step that must be performed in HS-SPME/GC-MS analysis is sample collection, which provides the matrix containing the VOCs to be extracted by HS-SPME. This matrix can be any one of a diverse set of materials, such as gauze, cotton, or glass beads, that can come into contact with the skin surface for a while and become impregnated by any VOCs responsible for skin odor. These can then be extracted by thermal volatilization or solvent extraction, followed by fiber exposure in an enclosed SPME system [1]. Other sampling methods can be used, such as the use of solvents directly on the skin or even scraping to obtain skin tissue samples and, as with other odor sampling methods, the collection of fresh sweat [9,10]. A less invasive method than the last method is the timed exposure of an SPME fiber directly onto the skin surface for VOC collection [1,11].

Although each one of the aforementioned skin odor sampling methods was applied successfully in previous studies, all of them have some significant limitations. For example, the matrices can be biologically sterile (except the skin itself) without being chemically selective, allowing for the detection of other compounds as well as those that are truly responsible for skin odor [11]. The direct use of fibers has the advantage of not requiring any steps prior to GC-MS analysis (the HS-SPME step can be skipped), as the fibers that are usually used in the HS-SPME step can be directly inserted into this system after VOC sampling [12]. Nevertheless, such a sampling method probably limits the number of sampled subjects at the same time, as it would require a fiber for each individual, which would both raise the sampling costs per individual and demand a long period of time to sample all subjects. In the event that there are a limited number of fibers, it would be necessary to clean the fiber between odor collections, requiring a laboratory setting with a GC instrument, which would be impractical to have in many field areas, including rural zones. The use of skin tissue can be considered an invasive method because there is some risk of accidental cuts. The use of solvents can also be invasive, as it can evoke an allergic reaction in the individual.

Furthermore, all of these sampling methods often require a long time to be applied, as they involve several steps and can demand volunteers to spend a long period of time collecting samples of their own skin odor (e.g., with Petri dishes, glass beads, gauze, or cotton, which frequently require solvent extraction as the next step), or expose areas from their body for the application of organic solvent directly onto skin (which can cause allergic reactions or skin irritation) and, in some cases, the volunteers may need to perform exercises in order to allow for sweat sampling [1,9,13,14,15]. These limitations are certainly inconvenient for studies with large populations, especially for field-scale studies.

We observed the need for an alternative method for field-scale human skin odor sampling that could be performed in a short amount of time with low-cost materials. We wanted a method offering comfortable sampling conditions with very low or no risk of adverse reactions or accidents to the volunteers. Additionally, as a way to reduce the risks of sample contamination, an appropriate alternative method would use a matrix that could be easily transported to and processed in a laboratory without the need for pretreatment prior to fast analysis by HS-SPME/GC-MS.

Focusing on finding a sampling method and a suitable matrix to satisfy the aforementioned characteristics, it was noted that many studies developed in the forensic science field used human hair as a biological matrix for VOC analysis by HS-SPME/GC-MS to find chemical markers that indicate the use of drugs, such as cocaine, marijuana, and alcohol [16,17,18,19]. Hair from dogs was also used as a biological matrix for HS-SPME/GC-MS analysis to detect VOC markers of *Leishmania infantum* infection [20,21]. To our knowledge, there are no reports about the use of human hair as a matrix in HS-SPME/GC-MS for the determination of the human skin odor profile. In the present study, we considered that human hair acts as retention mesh for human skin volatiles and, hence, can be used as a matrix for VOC extraction in HS-SPME/GC-MS analysis. Thus, we intend to investigate whether human hair can be used as a matrix for skin odor collection and HS-SPME/GC-MS analysis, with no need for pretreatment prior to analysis. This method would be a low-cost alternative with very low risk to the physical integrity of the investigated individual to current VOC sampling methods and, thus, can be considered a suitable method for field sampling.

## 2. Materials and Methods

### 2.1. Pilot Test

A pilot test was performed under laboratory conditions with the unique purpose of verifying if it would be possible to use human hair with HS-SPME/GC-MS to search for VOCs attributed to human skin odor. Considering that human hair can act as a retention mesh for VOCs emanated from human skin and that the human skin odor profile can be very diverse depending on the methods applied, we searched for the most common VOCs related to human skin odor. According to the literature, there are five VOCs that are frequently reported as present in human skin odor (see item c), regardless of the methods applied. Therefore, we determined that if these VOCs can be found in this pilot test, hair samples can be as suitable for skin VOC sampling as other sampling methods, including cotton pads or gauze, but with advantageous results, such as the fact that hair is in natural contact with the volunteer’s skin. Thus, the pilot study focused on detecting these five VOC markers as described below.

*(a)*  *Sample Collection*

Samples of human hair were collected under laboratory conditions from a thirty-year-old healthy male volunteer who was asked to avoid spicy food, alcohol, and smoking. He was also directed to not take a shower or use any soap or lotion on the skin during the twenty-four hours prior to sampling. Hair from the leg was sampled, as the leg is generally more hairy than other parts of the body and thus requires a smaller area for the acquirement of the same amount of hair. Moreover, volunteers would probably feel more comfortable donating hair from their legs because these parts of the body can be easily covered by clothes and call less attention than other parts, such as the arms. Three samples of 100 mg of hair were collected from the volunteer´s legs using portable hair clippers. The portable hair clippers were cleaned with 70% ethanol and air dried after each sampling procedure. The cuts measured between 1 and 3 mm in height from the human skin surface and were taken below the knees (the sampled area was limited from below the knee to the top of the ankle) from intact skin areas. Each sample was placed in a 20 mL glass vial sealed with an aluminum cap and silicone septum (all these items were purchased from Sigma-Aldrich, São Paulo, Brazil).

*(b)*  *SPME-HS/GC-MS Procedures*

Each glass vial containing 100 mg of hair was placed in an aluminum block (height = 4 cm; diameter = 8 cm), which was placed on a hot plate with a controlled temperature of 80 °C. A 65 μm polydimethylsiloxane/divinylbenzene (PDMS/DVB) fiber (Supelco, Bellefonte Sigma-Aldrich, Brazil) was inserted through each vial’s silicone septum with manual holder support for SPME and was exposed to the headspace sample for 45 min. After this, the fiber was recalled inside the manual holder. The fiber was then exposed to the GC-MS system (Shimadzu GC-2010/QP–2010 high performance quadrupole, Kyoto, Japan) according to the following instrumental conditions: The DB-1 MS capillary column was 30 m × 0.25 µm i.d. × 0.25 um film thickness (Agilent, Palo Alto, CA, USA); 3 min of thermal desorption; Helium (He) (>99% purity and supplied by White Martins, Goiânia, Brazil), flow rate of 0.7 mL min^−1^; oven temperature program: 40 °C for 3 min, 2 °C min^−1^ until 130 °C, 130 °C for 15 min, 2 °C min^−1^ until 205 °C, and 20 °C min^−1^ until 250 °C, 250 °C for 2 min; 25 kPa pressure; total flux of 5 mLmin^−1^; line velocity of 30.2 cm/s; 3 mL min^−1^ purge flux; the injector was used in “splitless” mode and its temperature was 240 °C; the temperature of the transfer line and ion source were also 240 °C; the electron impact energy was 70 eV and the scanning frequency was 2 s^−1^ from 50 m/z to 350 m/z. Peak detection was performed using GCMS Solution software (version 2.53, Shimadzu), considering the following parameters: The peak width at half height, Pw50, was 0.6 m/z units, as measured for the fragment ions at 40 m/z and 400 m/z from the mass spectrometer calibration standard perfluoro-tri-butylamine (PFTBA).

*(c)*  *Data Analysis*

The evaluation of hair from legs as a potential matrix for skin odor collection was based on chromatographic analysis for the detection of the VOCs 6-methylhept-5-en-2-one, octanal, nonanal, decanal and (5*E*)-6,10-dimethylundeca-5,9-dien-2-one (hereafter referred to as geranylacetone). These compounds were selected as quality markers because they are frequently reported in the literature as common VOCs of human skin odor [1]. 

### 2.2. Standardization of HS-SPME Method Parameters for Human Hair Analysis

Once the quality markers were detected in the pilot test (see Section 2.1), it was assumed that leg hair could be used for the investigation of the human skin odor profile. Thus, it would be necessary to optimize the analysis conditions. For this reason, the next step was to define which HS-SPME/GC-MS conditions would best meet our goals. The following parameters were considered for HS-SPME standardization: (a) Fiber type; (b) fiber exposure temperature for extraction; (c) fiber exposure time for extraction; (d) optimal amount of hair mass to be used as a matrix for VOC extraction. 

The instrumental conditions for GC-MS were the same as those used in the pilot test. Hair samples were collected from the same volunteer from the pilot test. All assays were performed in triplicate. Steps a, b, and c were accomplished using 100 mg of hair for each sample, while step d was performed to elect the appropriate mass of hair that would be suitable for analysis; thus, different amounts were tested, as explained below. Samples were stored at −20 °C until two hours prior to analysis.

*(a)*  *SPME Fiber Selection*

Three different types of fibers were tested to evaluate their efficiency for VOC extraction from human hair samples: 65 μm polydimethylsiloxane/divinylbenzene (PDMS/DVB), 75 μm Carboxen/ polydimethylsiloxane (CAR/PDMS) and 100 μm polydimethylsiloxane (PDMS) (Supelco, Bellefonte, PA, USA). Each fiber was exposed to the sample headspace for 45 min at 80 °C.

*(b)*  *Extraction Temperature*

HS-SPME was performed using a PDMS/DVB fiber which was exposed for 45 min and the temperatures evaluated were 40 °C, 50 °C, 60 °C, 70 °C, 80 °C, and 90 °C.

*(c)*  *Extraction Time*

VOC extractions in the HS-SPME system were evaluated during five different fiber exposure time intervals: 10, 20, 30, 40, and 50 min. All analyses were performed using a PDMS/DVB fiber at 90 °C.

*(d)*  *Amount of Hair for Sample (mg)*

For this step, a PDMS/DVB fiber was exposed for 40 min at 40 °C to the headspace of samples containing 20 mg, 50 mg, 100 mg, and 200 mg of hair.

*(e)*  *Data Analysis*

The efficiency of each tested parameter was analyzed by a one-way ANOVA test followed by Tukey´s multiple comparison post hoc test, considering the total number of average peaks obtained by each tested condition and the total average peak areas (relative intensity of VOCs present in the sample) of the observed peaks in the chromatograms. Nevertheless, considering that the amount of hair can eventually be a restrictive parameter for analysis, as some people may have sparse hair or can deny the donation of too much hair, the amount of mass to be used for each sample was defined based only on the total number of peaks detected in the chromatograms. The total area of VOC peaks is directly influenced by the amount of mass contained in each sample; thus, this aspect was not considered for this mass amount determination. The chromatogram analysis and the statistical analysis were performed using GCMS Solution software, version 2.53 (Shimadzu) and GraphPad Prism software, version 5.0 (GraphPad Software, Inc., San Diego, CA, USA—www.graphpad.com).

### 2.3. Analysis of Human Hair as a Matrix for Skin Odor Profile Determination Using HS-SPME/GC-MS—Laboratory and Field Applicability

*(a)*  *Laboratory Sampling and HS-SPME/GC-MS Conditions*

Hair from the legs of three healthy male volunteers (age 22–35 years, all residing in Salvador City, Bahia State, northeast Brazil) were collected (according to methods described in Section 2.1, subtopic “a”) and triplicates containing 20 mg (defined after the analysis of standardization test results, see Section 3.2) of hair were analyzed under the following conditions: HS-SPME was performed using a PDMS/DVB fiber at 90 °C during an exposure time of 40 min; GC-MS conditions were the same as used in the pilot test. These parameters were determined according to the results obtained in the standardization steps.

*(b)*  *Volatile Organic Compound (VOC) Identification*

Identification of VOCs was achieved by (i) comparing the GC retention times and mass spectra with those of the pure standard compounds, when available, (ii) comparing all mass spectra with the data system library (NIST 08 database), and (iii) determining Linear Retention index (LRI) values by the equation proposed by van den Dool and Kratz [22] using a homologous series of *n*-alkanes C8–C40, injected directly in the GC injector under the same chromatographic conditions as the samples. These values were compared with values reported in the literature (NIST Chemistry WebBook—webbook.nist.gov) for similar chromatographic columns. A standard solution of hydrocarbons and the synthetic standard compounds (highlighted in Table 1), with purities of 99% or higher, were purchased from Sigma–Aldrich, São Paulo, SP, Brazil.

*(c)*  *Data Analysis*

To verify whether human hair obtained from legs could be used as a matrix for skin odor extraction and detection via HS-SPME/GC-MS, the detected VOCs were compared with those that were attributed to human skin odor as found in previous studies utilizing a variety of methods.

### 2.4. Field Applicability of the Standardized Method for Skin Odor Collection and Laboratory Analyses

*(a)*  *Field Sampling*

Field sampling was performed during a work week in Corte de Pedra, 275 km from Salvador City, located in the municipality of Tancredo Neves, southeastern Bahia State in the Brazilian northeast. The procedures were performed according to Section 2.1 and at least 60 mg of hair from the legs was collected and stored at −20 °C until the moment of laboratory analysis in Salvador, Bahia, Brazil. A total of 99 samples of leg hair was collected from 33 healthy male volunteers between the ages of 18 and 60.

*(b)*  *Laboratory Analysis*

The results obtained from the HS-SPME/GC-MS analysis were then submitted for VOC identification, which was performed following the steps described in Section 2.3.

*(c)*  *Ethics Statement*

This study was approved by the Institutional Review Board of the Gonçalo Moniz Institute, Oswaldo Cruz Foundation (protocol number 033030/2015), and all participants were volunteers who had signed a term of free informed consent prior to their participation in this study.

*(d)*  *Data Analysis*

The field applicability of the standardized method for human skin odor profile determination was evaluated as described in Section 2.3, item c. The results obtained in the present section were also compared to those obtained in Section 2.3. 

## 3. Results

### 3.1. Pilot Test

**Figure 1:**
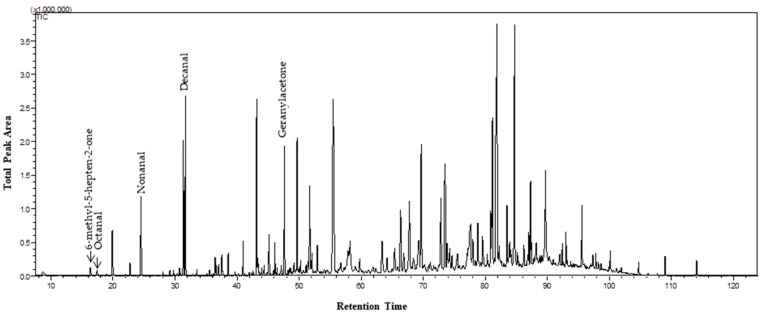
Total ion chromatogram showing the five peaks of volatile organic compounds selected as quality markers, detected as a function of retention time.

All five quality markers (6-methyl-5-hepten-2-one, octanal, nonanal, decanal, and geranylacetone) were detected in the pilot test, which indicated that hair from human legs could be used to collect VOCs from human skin and thus be a matrix for the extraction of these VOCs by HS-SPME/GC-MS (Figure 1). 

### 3.2. Standardization of HS-SPME Method Parameters for Human Hair Analysis

**Figure 2:**
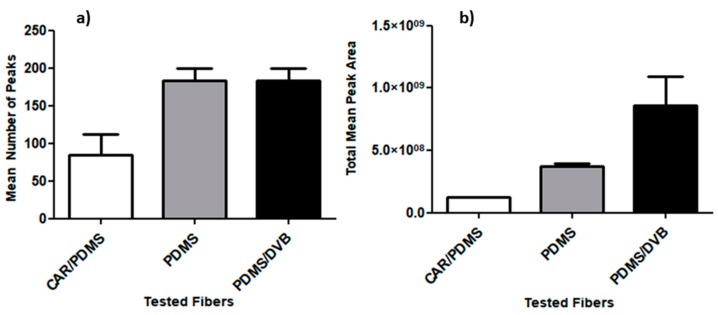
(**a**) Mean of the total number of volatile organic compound peaks and (**b**) of the total peak area of volatile organic compounds detected via chromatographic analysis using the tested fiber types during the solid-phase microextraction step. Carboxen/polydimethylsiloxane (CAR/PDMS), polydimethylsiloxane (PDMS), polydimethylsiloxane/divinylbenzene (PDMS/DVB).

According to the one-way ANOVA with Turkey’s post hoc test, both the 100 μm PDMS and 65 μm PDMS/DVB fibers extracted significantly more VOCs than the CAR/PDMS fiber in SPME-HS/GC-MS analysis (*P* = 0.0245; *n* = 3). In fact, the PDMS and PDMS/DVB fibers extracted the same number of peaks (Figure 2a), but the PDMS/DVB fibers yielded a higher total peak area (*P* = 0.0188; *n* = 3) (Figure 2b) and thus were chosen as the best type of fiber for VOC extraction. 

**Figure 3:**
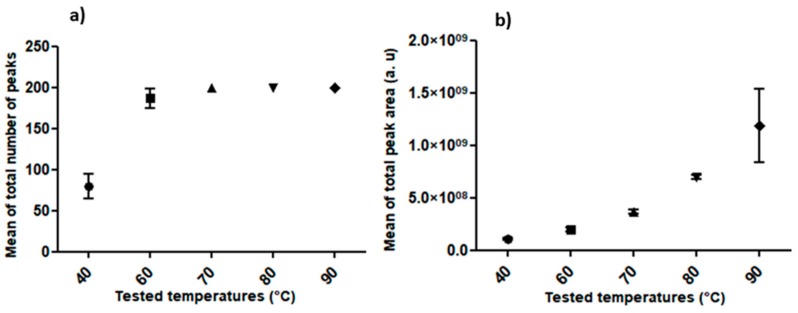
(**a**) Mean of the total number of volatile organic compound peaks and (**b**) of the total area of volatile organic compound peaks detected via chromatographic analysis using a PDMS/DVB fiber under different temperatures (40 °C, 50 °C, 60 °C, 70 °C, 80 °C, and 90 °C) for 40 min during the solid-phase microextraction step.

Under each tested temperature, 79 and 200 peaks were obtained, with higher numbers associated with the temperature increase. Greater numbers of VOCs were achieved at 60 °C (*n* = 186), 70 °C, 80 °C, and 90 °C (*n* = 200), which were significantly higher than the number of peaks obtained at 40 °C (*P* = 0.0006; *n* = 5). Although no significant differences were observed among the total peak areas obtained at 70 °C, 80 °C, and 90 °C, the total area was directly influenced by the temperature rise. Thus, 90 °C was defined as the temperature for VOC extractions in the next steps (Figure 3). 

**Figure 4:**
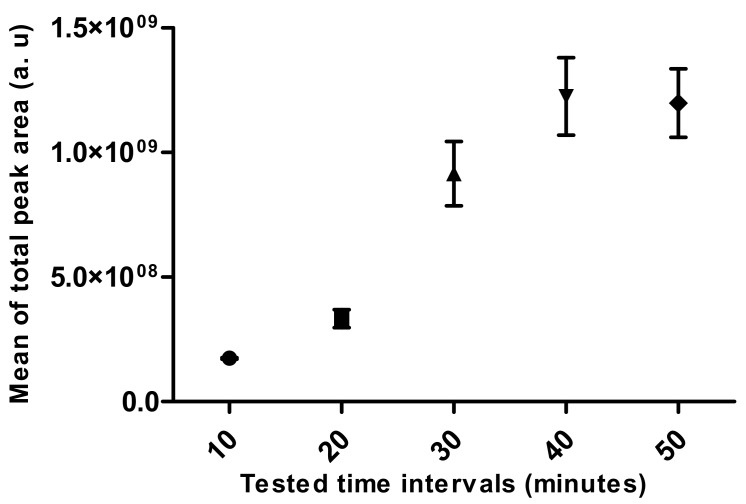
Mean of the total peak area of volatile organic compounds obtained via chromatographic analysis using a PDMS/DVB fiber at different time intervals (10, 20, 30, 40, and 50 min) in the microextraction step.

Although the same number of peaks (*n* = 200) was obtained for each tested time interval, the highest peak area was obtained after 40 min of fiber exposure (*P* = 0.003; *n* = 5) (Figure 4). As the last step, the amount of mass per sample was evaluated and the number of obtained peaks was the same for all tested masses (*n* = 150). As the total peak area was not relevant to define this parameter, the lower mass (20 mg) was defined as the appropriate one for all tests. 

### 3.3. Analysis of Human Hair as a Matrix for Skin Odor Profile Determination by HS-SPME/GC-MS

**Figure 5:**
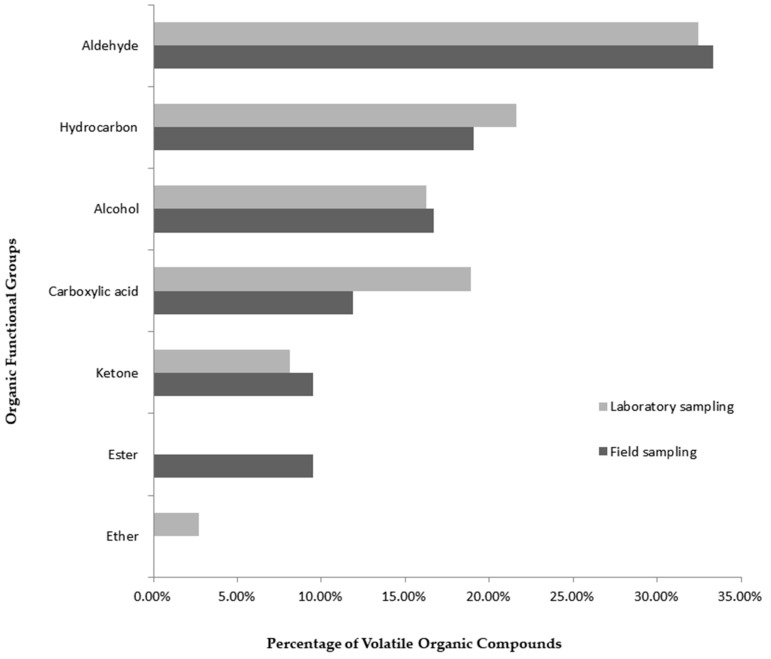
Comparison between the percentages of volatile organic compounds, distributed among seven different organic functional groups, obtained from the HS-SPME/GC-MS analysis of samples collected under laboratory and field conditions.

A total of 37 VOCs, including the five quality markers, were identified in the nine hair samples collected from three volunteers (Table 1). These samples were obtained for the investigation of the laboratory applicability of the use of human hair as a matrix for skin odor analysis by HS-SPME/GC-MS. The identified VOCs belonged to seven different classes of organic compounds: Alcohols, aldehydes, carboxylic acids, ketones, esters, ether and hydrocarbons. Twelve out of the 37 identified VOCs (32.43%) were classified as aldehydes, followed by 8 (21.62%) classified as hydrocarbons, 6 (16.22 %) classified as alcohols, 7 (18.92%) classified as carboxylic acids, 3 (8.11%) classified as ketones, and 1 (2.7%) classified as an ether (Figure 5). 

### 3.4. Field Applicability of the Standardized Method for Skin Odor Profile Determination and Laboratory Analyses

The evaluation of the field applicability of the standardized method for skin odor collection and its laboratory analysis was performed using 99 hair samples obtained from 33 volunteers from Corte de Pedra, Bahia. A total of 42 VOCs were identified, including the quality markers (Table 1). These VOCs can be distributed among six functional groups: Aldehydes (14/42; 33.33%), hydrocarbons (8/42; 19.05%), alcohols (7/42; 16.67%), carboxylic acids (5/42; 11.9%), ketones (4/42; 9.52%), and esters (4/42; 9.52%), as shown in Figure 5. The percentage of VOCs belonging to each of these functional groups was similar for laboratory and field sampling (*P* = 0.9988; Df = 5).

## 4. Discussion

By applying the same sampling method and method for VOC extraction and analysis, the present study identified 37 VOCs from three volunteers residing in an urban area (Salvador City) and 42 VOCs from 33 different subjects residing in a rural area 275 km from Salvador, reaching a total of 49 identified compounds. It is important to observe that studies in which the extraction step was performed in a confined space, as in headspace solid-phase microextraction with no external interferences (e.g., surrounding air) resulted in the detection of 20–90 VOCs [8,9,24,25,26]. Therefore, the resulting 49 identified compounds acquired in the present study are in accordance with this interval.

Previous studies have reported high quantitative and qualitative variability of human skin VOCs. Such variability has been associated with a diversity of factors, such as the region of the body that is considered for analysis and the methods applied to investigate the skin odor profile [1], although the sampling method is presumed to be the largest factor. Indeed, different methods and different numbers of subjects have been utilized in several studies, as outlined in this section.

As mentioned above, the methods adopted in the present study resulted in a total of 49 detected VOCs, while more than 400 VOCs were already detected and identified as released from human skin odors. However, until now, the highest numbers of VOCs detected were achieved by combining different methods or by including a large number of subjects, as reported by Bernier, Booth, and Yost and Penn and colleagues, whose studies resulted in 303 VOCs detected by using glass beads exposed to skin for a few minutes by four different volunteers and 373 VOCs detected by exposing PDMS-coated stir bars to the sweaty armpit surfaces of 197 volunteers [13,14]. Glass beads were also applied by Verhulst and colleagues by rubbing these beads against the feet of 48 volunteers, which resulted in the identification of 15 VOCs [5]. The methods applied by Bernier, Booth, and Yost and by Verhulst and colleagues required approximately 10–15 min of rubbing glass beads to collect the skin VOCs [5,13], which can be unpleasant for the volunteers and demand a long time for sampling. The 373 VOCs reported by Penn and colleagues, however, were obtained by collecting samples five times from each of the 197 volunteers that participated in this study, which also required a great deal of time and effort by the researchers [14].

Another study that may be relevant to be mentioned here was produced by Dormont and colleagues, who investigated the human skin VOCs obtained by four different methods, from a total of 26 subjects: (1) Solvent extraction from blades used for scraping heels; (2) SPME fiber exposure upon feet enclosed inside plastic bags; (3) contact SPME, by directly placing the fiber upon the skin of the feet; (4) and using a dynamic headspace that functioned by enclosing the feet in a system that allowed for VOC adsorption by a polymer, such as a purge and trap system [9]. In this investigation, a total of 44 VOCs were detected, most of which were detected by all four methods. In another study, a PDMS membrane was directly applied to the back, forearm, and thigh of one unique individual and 99 VOCs were identified, 27 of which were detected in all three body areas [23].

All the methods mentioned here showed efficiency in collecting VOCs related to human skin odors. However, as observed in other studies that used, for example, sweat, gauze, cotton, or t-shirts exposed for periods of time (minutes, hours, and even several days) to collect VOCs, all of these approaches have many inconveniences. Among the major issues, we can cite the following: The use of organic solvents directly on skin, which can cause some tissue irritation; the use of blades for skin scraping, which represents a risk to the physical integrity of the individual (e.g., accidental skin cuts); the need to perform GC-MS analysis immediately after sample collection, which is not always possible; the need for pretreatments of sampling materials (e.g., gauze and cotton); the time required from the volunteers during sampling, which, depending on the method, can vary from a few minutes to hours or days and can even require that the subject perform some physical exercise to produce sweat prior to sampling [5,8,9,13,15,23,25,26,27,28]. 

The present study obtained relevant results with practically no inconveniences. In addition, the numbers of VOCs detected by the methods applied in the present study (37 and 42 from urban and rural samplings, respectively) were in accordance with those described in the literature. All five adopted quality markers, considered the minimum requirements for our purpose (6-methyl-5-hepten-2-one, octanal, nonanal, decanal, and geranylacetone), as they are considered the most common VOCs found in human skin, were also detected [1]. We also verified that 73.5% of these identified VOCs (total of 49) were already reported in previous studies. Considering laboratory and field sampling separately, 78.37% and 83.3%, respectively, of the identified VOCs were already reported in other studies (Table 1). Furthermore, the chemical analysis also demonstrated that all the identified VOCs were within the chemical functional groups described by Dormont et al. [1] as the most common in human skin odors, which included carboxylic acids, aldehydes, ester derivatives, hydrocarbons (mainly alkanes), short chain alcohols, and ketones [1].

Finally, these findings support the claim that SPME-HS/GC-MS analysis of hair from legs is a suitable method for human skin odor profile investigation. Similar quantitative and qualitative results compared to those previously reported in the literature were obtained. It should be noted that the results from literature studies were obtained by various expensive and usually laborious methods that were more difficult to apply in field sampling than what our method demanded. In other words, the present study achieved the detection of a very similar number of compounds using a unique sampling method, applied in only one part of the body and without using any pretreatment prior to SPME analysis. This allows for a simple, fast, and affordable method for skin odor VOC collection. 

Of course, every method has its limitations and we have no pretensions that we developed a perfect method for human skin odor analysis. Indeed, hair sampling can be a limitation when including female volunteers, as they commonly shave their legs, so requesting the allowance of hair growth can easily be denied by female volunteers. Nevertheless, this limitation seems to be insignificant considering all the advantages compared to other methods. Therefore, the presented method is a suitable alternative for skin odor collection and hair from legs is a suitable matrix for VOC extraction and detection using SPME-HS/GC-MS. This method is a fast, easy, low-cost, and safe method for human skin odor studies, especially for field studies.

## 5. Conclusions

Human skin odor studies are a very complex task. The existing literature clearly confirms that there is a large variety of methods in place for such investigations and none of them seemed to be a suitable option for field studies. The hypothesis that hair from legs can be utilized as an efficient tool for human skin VOC sampling was confirmed. The method that we proposed can be considered suitable and practical for skin odor profile determination, with no need for pretreatment prior to analysis, relatively low cost, and minimum discomfort and practically no risk for participants.

## Figures and Tables

**Table 1:** List of the identified volatile organic compounds (VOCs) obtained by SPME-HS/GC-MS analysis of hair samples acquired during laboratory sampling and field sampling.

VOC	Nomenclature (IUPAC)	RT (min)	Functional Group	LRI_exp._	LRI_lit._	Lab. Samp.–Content (%)	Field Samp.–Content (%)	CSASI	References
1	**6-methyl-5-hepten-2-one**	16.10	Ketone	962	963	0.21	0.55	X	[4], [6], [7], [10], [13], [23]
2	**Octanal**	17.15	Aldehyde	978	978	0.27	0.51	X	[4], [6], [7], [9], [10], [13], [24]
3	**2-phenylacetaldehyde**	18.68	Aldehyde	1002	1002	0.21	0.04	X	[15]
4	**1-octanol**	22.42	Alcohol	1055	1055	0.03	0.06	X	[4], [9], [13]
5	6-methyl-3,5-heptadien-2-one	23.74	Ketone	1074	1074	0	0.05		[13]
6	**Nonanal**	24.21	Aldehyde	1081	1081	1.36	6.59	X	[4], [6], [7], [9], [10], [24]
7	*Cis*-verbenol	27.00	Alcohol	1121	1121	0	0.02		-
8	**2-nonenal**	27.76	Aldehyde	1131	1130	0.08	0.77		[4], [6], [9]
9	**1-nonanol**	29.50	Alcohol	1156	1157	0.12	0.27	X	[4], [10], [13]
10	Verbenone	30.42	Ketone	1169	1176	0	0.42	X	-
11	**Decanal**	31.36	Aldehyde	1183	1183	3.87	8.90	X	[4], [6], [9], [10], [13], [24]
12	2-decenal	34.85	Aldehyde	1234	1236	0	0.24		[4], [6]
13	Decanol	36.29	Alcohol	1255	1255	0.20	0		[9]
14	**Nonanoic acid**	36.91	Carboxylic acid	1264	1268	0.04	1.06	X	[6]
15	2-undecanone	37.43	Ketone	1272	1272	0.23	0		-
16	**Undecanal**	38.25	Aldehyde	1284	1286	0.41	0.99	X	[4], [10]
17	**2-undecenal**	41.63	Aldehyde	1336	1340	0.08	0.49		[10]
18	Decanoic acid	43.33	Carboxylic acid	1362	1359	0.27	0		-
19	**Dodecanal**	44.84	Aldehyde	1386	1386	0.38	1.02		-
20	**Tetradecane**	45.75	Hydrocarbon	1400	1400	0.28	0.34	X	[2], [4], [6]
21	Gamma decalactone	46.94	Ester	1418	1415	0	0.08		-
22	Methylparaben	47.19	Ester	1421	1420	0	0.31		-
23	**Geranylacetone**	47.30	Ketone	1423	1423	0.99	2.92		[6], [7], [25]
24	**Dodecen-1-al**	48.10	Aldehyde	1435	1442	0.20	0.31		-
25	**1-dodecanol**	49.32	Alcohol	1453	1456	1.87	0.66	X	[6], [10], [13]
26	Tridecanal	51.47	Aldehyde	1485	1488	0	0.51		[6]
27	**Pentadecane**	52.44	Hydrocarbon	1500	1500	0.37	1.18	X	[2], [4], [6], [10]
28	**(*E*)-2-tridecenal**	55.97	Aldehyde	1535	1541	0.23	0.16		[6]
29	**Dodecanoic acid**	57.47	Carboxylic acid	1550	1556	1.75	1.67		[2], [4], [7], [10]
30	**Tetradecanal**	61.17	Aldehyde	1587	1588	0.11	0.28		[4], [10]
31	**Hexadecane**	62.55	Hydrocarbon	1600	1600	0.94	1.07	X	[4], [6]
32	Octyl ether	67.78	Ether	1651	1657	0.63	0		-
33	**1-tetradecanol**	69.09	Alcohol	1664	1664	4.97	6.25	X	[2], [10], [13]
34	**Pentadecanal**	72.02	Aldehyde	1692	1693	2.56	4.89		[10]
35	**Heptadecane**	72.90	Hydrocarbon	1701	1700	1.30	1.00	X	[2], [4], [6]
36	6-phenyldodecane	73.82	Hydrocarbon	1713	1719	0.37	0		-
37	**Tetradecanoic acid**	77.57	Carboxylic acid	1763	1762	3.12	1.33	X	[2], [7], [10], [24]
38	2-ethylhexyl salicylate	78.32	Ester	1773	1769	0	2.26	X	[7]
39	Ethyl myristate	78.88	Carboxylic acid	1780	1778	0.74	0		-
40	2-phenyl dodecane	79.20	Hydrocarbon	1785	1794	0	0.16		-
41	**Octadecane**	80.41	Hydrocarbon	1801	1800	1.81	1.78	X	[10]
42	Pentadecanoic acid	83.64	Carboxylic acid	1853	1860	0	0.80		[2], [7]
43	**1-hexadecanol**	84.31	Alcohol	1864	1866	10.59	21.13	X	[2], [9], [13]
44	**Nonadecane**	86.59	Hydrocarbon	1901	1900	0.38	2.17	X	[10]
45	Methyl hexadecanoate	87.08	Carboxylic acid	1910	1909	0.48	0		-
46	**Hexadecanoic acid**	89.56	Carboxylic acid	1955	1956	2.25	2.57		[2], [7], [10]
47	**Eicosane**	92.03	Hydrocarbon	2001	2000	0.96	2.43	X	[10]
48	Isopropyl palmitate	92.62	Ester	2013	2017	0	8.33		[10]
49	1-octadecanol	95.26	Alcohol	2066	2070	0	13.43	X	[9]

RT: Retention Time; LRI_experimental_: Linear Retention Index obtained experimentally; LRI_literature_: Linear Retention Index obtained from literature (NIST Chemistry WebBook—webbook.nist.gov); CSASI: Confirmed with synthetic analytic standard injection. The text in bold highlights the compounds detected in the skin from both urban and field volunteers.
